# Abuse of the Eyes

**Published:** 1870-10

**Authors:** 


### The Bistoury.

ELMIRA, N. Y., OCT., 1870.

The BISTOURY is published Quarterly, upon the 1st o April, July, October and January, at Fifty Cents a year in advance.

Club Rates :--$25, will be paid for every 100 subscribers.

### ABUSE OF THE EYES.

There are those who, in consequence of a feeble constitution, or a highly susceptible nervous organization, are wholly unable to engage in reading, writing, sewing, or other employments of this character, without producing functional disturbance of, and experiencing pain in the eyes, followed by considerable congestion or a "blood-shot" appearance of the eye-ball. Such persons in reading, will frequently stop to press their fingers upon their eyes in order to relieve a tired feeling which prevails about them, and are often compelled to bathe their eyes in cold water to relieve them from a sense of heat which takes possession of them when the reading is protracted. To persist in any of these employments, when the person knows to a certainty that harm must result to the eyes, is of course wrong, but it becomes a difficult task for us to present any argument that will induce persons so inclined to abandon such practices; our experience with them has been that they continue in it until vision becomes so imperfect as to debar them from reading or writing entirely, when they at once complete their blindness by *trying* to read by aid of magnifying lenses and artificial light. Persons in good health too, often have the same symptoms after incessant reading in a finely printed book, reading on the cars, sewing upon black work by lamp light, preparing long manuscripts, working at watch work, *etc.* If they persist in their work, pain is produced in the eye itself, in the orbit and over the brow, and reflected light from snow or the side-walk upon a bright day, becomes excessively painful and almost intolerable. The eye gradually becomes inflamed, and tears flow from them profusely, the person is said to have "weak eyes," when everybody, together with everybody's mother, will recommend the application of all manner of washes, lotions, salves and poultices, until the unfortunate patient is "doctored" blind. This disease appears frequently among those who are forced to sew and stitch by artificial light, in order to sustain themselves and their families. When they know their eyes are suffering they cannot afford to stop, for by so doing bread stops also, and so they are compelled to work until vision is nearly lost. Much trouble is experienced in the treatment of such cases, for the reason that the most beneficial portion of the treatment, rest, cannot be afforded, and without it, no treatment, however skillful, can avail. Change of diet and residence, or a sea voyage, to those who can afford it, and entire suspension from any employment in which the use of the eyes will be required, are positively demanded.

When you find any of the above described symptoms coming on while reading or working, quit at once—bathe the eyes freely in cold water, and walk in the open air, and should not this restore the healthy action of your eyes, cease to be actively employed and secure means for invigorating your general health. All labor by artificial light should be avoided, as well as visiting highly illuminated and badly ventilated apartments. Densely crowded rooms should also be shunned. Ladies, whose eyes are "weak," should put away gaudy worsted works, or needle work upon bright colored materials—should walk daily from their furnace-heated apartments at home, and astonish their lungs with a breath of fresh air—should bathe the eyes two or three times in the middle of the day, with salt water—should keep their gas-lights shaded, and a window in every room in the house with two inches of open space at the top—eat less confectionery and more roast beef and graham bread—retire by nine at night, and arise by six in the morning, and walk twenty minutes in the open air before breakfast;—if they like the treatment, and find that it agrees with them, the probabilities are that no medical interference will be necessary.

There is another form of disease having symptoms closely allied to those just described, and caused by the same abuse of the eye, but is by far the most dangerous in its results. It will be familiar to most of our readers, under the general term of *amaurosis.* We find it most frequently among the more refined class of community, those who indulge in high living, fashionable parties, and novel reading or story writing, at an hour when most people are in bed. The symptoms ushering in this disease, are a haziness or dimness of vision, as though looking through a fog; threads, lines or strings of globules seem moving through the air; sparks, flashes or circles of fire are seen, especially at night, and a dull halo is seen about the gas-burner.—The patient sees best in a strong light, and a dull pain is felt through the head. These symptoms may be the result of overwork, but sometimes are not; they always, however, furnish evidence of the coming on of serious congestion of the *retina* or nervous portion of the eye, and require more active treatment than mere rest.

